# Percutaneous Reduction and Fixation for Traumatic Thoracolumbar Vertebral Fracture Using a Monoaxial Screw System

**DOI:** 10.7759/cureus.84815

**Published:** 2025-05-26

**Authors:** Shunsuke Kobayashi, Akira Shinohara, Tadashi Kimura, Shunsuke Katsumi, Mitsuru Saito

**Affiliations:** 1 Department of Orthopedic Surgery, Saitama Jikei Hospital, Saitama, JPN; 2 Department of Orthopedic Surgery, Jikei University School of Medicine, Tokyo, JPN

**Keywords:** ligamentotaxis, minimally invasive spine stabilization, monoaxial screw, percutaneous pedicle screw, thoracolumbar vertebral fracture

## Abstract

Background and aim: The development of percutaneous pedicle screws (PPSs) has led to the development of minimally invasive spine stabilization (MISt) procedures, which has decreased the invasiveness of spine surgery. Conventionally, PPSs were available as polyaxial screws only, making it technically challenging to perform percutaneous repair of thoracolumbar vertebral fractures with implants. This study aimed to evaluate percutaneous posterior fixation for thoracolumbar vertebral fractures using a monoaxial screw system and determine imaging and clinical outcomes until after implant removal.

Methods: We retrospectively reviewed 20 patients who underwent posterior fixation using the S4 Spinal Fracture Reduction Instrumentation (FRI) system with percutaneous vertebroplasty for traumatic thoracolumbar vertebral fractures. Follow-up continued until after implant removal. AO classification was type A3 in 19 patients and type B2 in one. Implants were removed after confirmation of bone healing. The mean follow-up period was 753 days. We evaluated intraoperative blood loss, operative time, neurological status, and radiological parameters, including local kyphosis angle, wedge deformity rate, and spinal canal stenosis rate.

Results: No surgical complications (e.g., worsening paralysis or infection) occurred. No patients had implant failure or required blood transfusion during surgery, reoperation, or routine painkiller use at the final follow-up. The mean spinal kyphosis angle was 12.6° preoperatively, 1.9° immediately postoperatively, 6.5° before implant removal, and 7.9° after implant removal, showing significant improvement between the preoperative and immediate postoperative periods. However, there was a significant loss of correction after implant removal compared with immediate postoperatively (mean 6°). Kyphosis angle tended to decrease from the preoperative period to after implant removal, albeit not significantly. The mean wedge deformity rate and spinal canal stenosis rate showed significant improvement immediately postoperatively. These improvements were maintained after implant removal.

Conclusions: Posterior fixation using the monoaxial PPS with the S4 Spinal FRI system is minimally invasive and is considered a useful surgical technique for thoracolumbar vertebral fractures.

## Introduction

Various methods have been used to treat thoracolumbar vertebral fractures, including conservative therapy, posterior fixation, anterior fixation, and combined posterior-anterior fixation. However, the choice of treatment remains controversial.

The development of percutaneous pedicle screws (PPSs) has advanced minimally invasive spine stabilization (MISt) procedures, reducing the invasiveness of spine surgery. Initially, PPSs were used for single-level posterior lumbar fusion or transforaminal lumbar interbody fusion, but they are now also used in various MISt-specific devices that allow for multivertebral fixation [1]. These devices have expanded the indications for MISt to include tumors, infections, and trauma [2,3].

Conventionally, PPSs were available only as polyaxial screws, making percutaneous repair of thoracolumbar vertebral fractures technically challenging. However, in 2011, B. Braun Aesculap AG (Melsungen, Germany) introduced the S4 Spinal Fracture Reduction Instrumentation (FRI) system, a monoaxial PPS system that enables percutaneous kyphosis correction and reduction with distraction. Monoaxial screws have the advantage of less loss of correction in comparison with polyaxial screws.

Although good outcomes have been reported for single-level posterior intervertebral fixation using Schantz screws and PPSs, there have been no studies evaluating the S4 Spinal FRI System alone for traumatic thoracolumbar vertebral body fractures [4-6]. This study retrospectively evaluates percutaneous posterior fixation using this system and reviews clinical and radiological outcomes until after implant removal.

## Materials and methods

Patients

Twenty patients (13 men and seven women) who underwent posterior fixation using the S4 Spinal FRI System (B. Braun Aesculap AG: Melsungen, Germany) with percutaneous vertebroplasty (PVP) for traumatic thoracolumbar vertebral fractures at Kashiwa Hospital, Jikei University School of Medicine, from August 2012 to March 2017 were followed up until after implants removal. The mean age at surgery was 44±16 (range: 23-73) years. The cause of injury included falls in 12 patients, sports activities in four, traffic accidents in three, and the impact of a heavy falling object in one. Osteoporotic vertebral fractures were excluded. Fracture levels were T11 in one patient, T12 in four, L1 in 11, L2 in three, and L3 in one. AO classification was type A3 in 19 patients and type B2 in one [7]. The mean load-sharing classification score was 6.0 (range: 4-7) [8]. Preoperative neurological deficits were observed in four patients, and the American Spinal Injury Association Impairment Scale (AIS) grade was C in one patient, D in three, and E in 16. Implants were removed after confirmation of bone healing on computed tomography (CT), with a mean postoperative period of 326±96 days. The mean follow-up period was 753 days. This study complied with the Strengthening the Reporting of Observational Studies in Epidemiology (STROBE) guidelines. This study was conducted in accordance with the Declaration of Helsinki and was approved by our institution’s research ethics committee. Informed consent was obtained from all individual participants included in the study.

Surgical procedure

To preserve the adjacent intervertebral space, fixation was limited to one level above and one level below the fracture. When necessary, polyaxial PPSs were combined with monoaxial PPSs to extend fixation in cases of small pedicle diameters or adjacent vertebral fractures.

Preoperative planning

**Figure 1:**
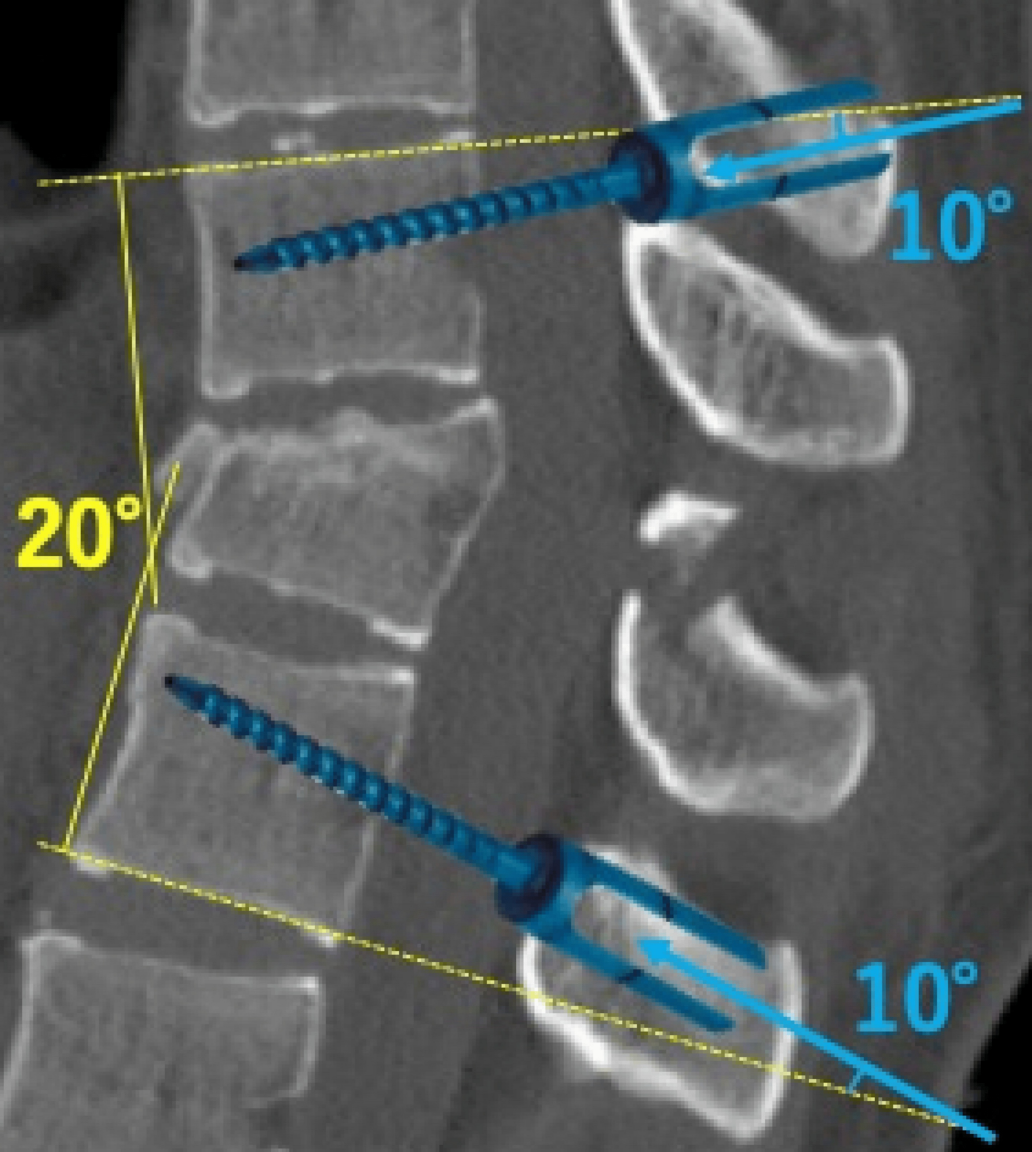
Insertion angle of monoaxial PPS. This case involves a fracture of the 12th thoracic vertebra with a local kyphosis angle of approximately 20°. To correct the kyphosis, we plan to insert the monoaxial PPS with a 10° cephalocaudal angulation. This image is created by the author (Shunsuke Kobayashi) of this study. PPS: percutaneous pedicle screw

Plain X-ray radiography and CT were used to determine the screw size and direction of penetration preoperatively. The longest and thickest screws possible were selected for correction of alignment. Since the monoaxial PPS fixes the screw at a 90° angle to the rod, the penetration direction was planned based on the anticipated alignment after kyphosis correction (Figure 1).

Approach

A longitudinal skin incision was made at the PPS insertion site to facilitate the attachment and removal of the FRI. For PVP, a small incision was made lateral to the point of PPS insertion to allow for rod insertion.

Insertion of monoaxial PPS and bone biopsy needle for PVP

First, a PPS probe was inserted into the vertebrae above and below the fracture. A bone biopsy needle for PVP was then inserted into the fractured vertebra, positioned at a slightly oblique angle to accommodate the subsequent rod insertion. The monoaxial PPSs were then inserted under fluoroscopic guidance using the conventional method. Finally, the rod was inserted percutaneously, taking care not to pinch the fascia.

Kyphosis correction

**Figure 2:**
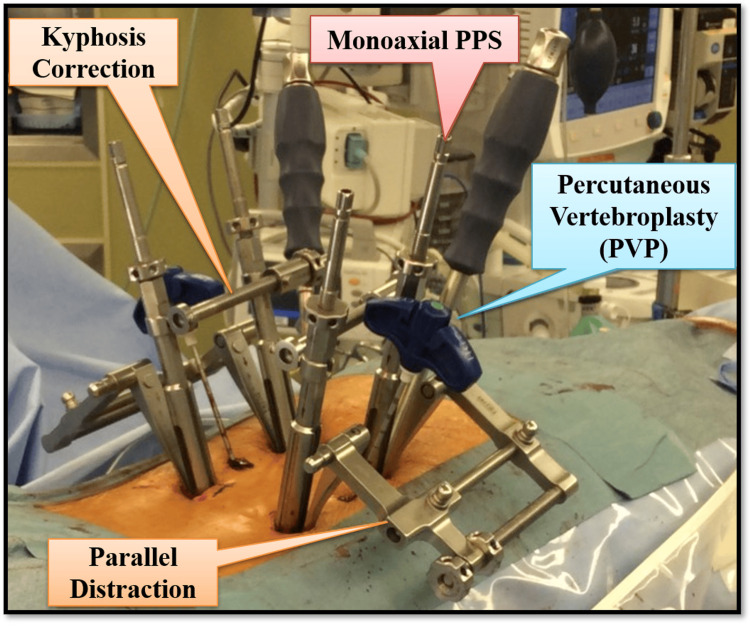
Fracture reduction instrument. Percutaneous kyphosis correction and reduction with ligamentotaxis after insertion of monoaxial PPS. The image is created by the author (Shunsuke Kobayashi) of this study. PPS: percutaneous pedicle screw

An FRI was installed, and kyphosis correction was performed carefully while monitoring the real-time fluoroscopic lateral view (Figure 2). The kyphosis was corrected by rotating the distally placed nut, although manual correction could also be achieved by grasping the upper end of the screw extender.

Parallel distraction

Parallel distraction was applied by rotating the proximally placed nut, reducing the fracture through ligamentotaxis. To prevent excessive distraction that could cause the disc space to widen, the real-time fluoroscopic lateral view was carefully monitored.

Percutaneous vertebroplasty

After final fixation, the FRI was removed. PVP was performed using the preplaced bone biopsy needle, with a hydroxyapatite (HA) block placed in the appropriate position under fluoroscopic guidance to fill the defect caused by the endplate fracture and kyphosis correction. Bone grafting was not performed to preserve motion after implant removal.

**Figure 3:**
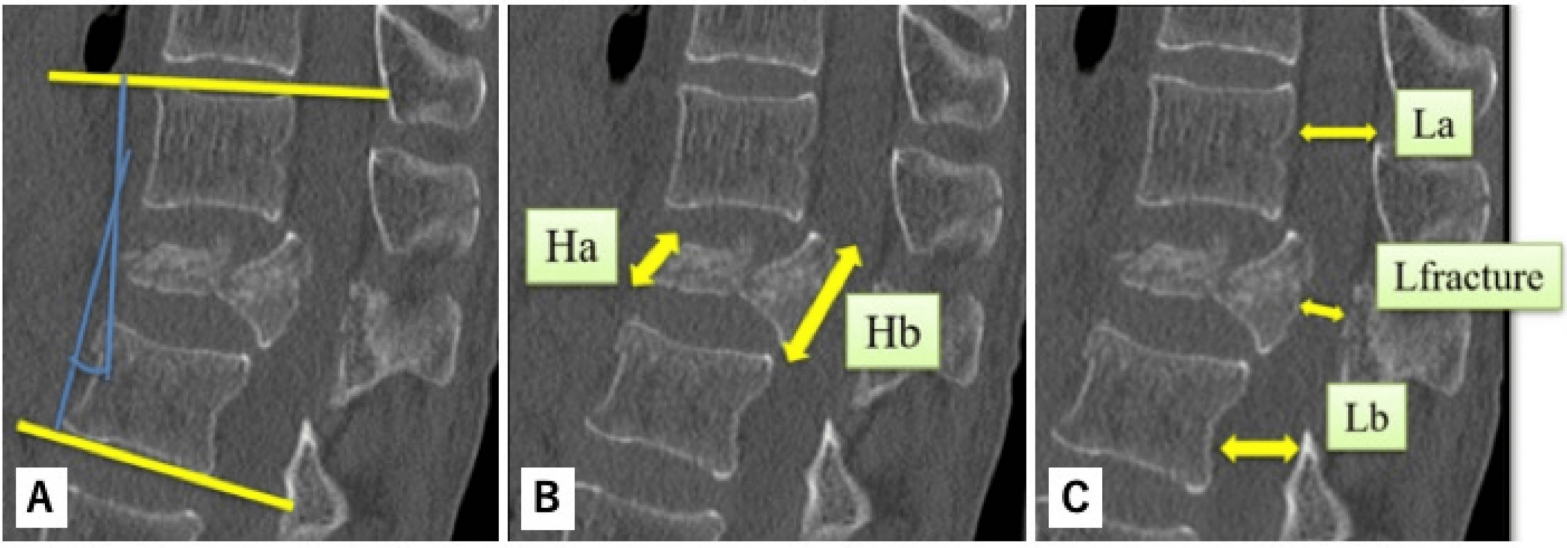
Radiological assessments using computed tomography (CT). (A) Local kyphosis angle. (B) Wedge deformity rate. (C) Spinal canal stenosis rate. Ha: Height of the anterior wall of the fractured vertebra; Hb: Height of the posterior wall; Lfracture: anterior-posterior diameter of the spinal canal at the fracture site; La: diameter at the cephalad vertebra; and Lb: diameter at the caudal vertebra. The image is created by the author (Shunsuke Kobayashi) of this study.

The following were determined preoperatively, immediately postoperatively, immediately before implant removal, and after implant removal (mean time to removal 304 days): intraoperative blood loss, operative time, neurological assessments, and radiological assessments using computed tomography (CT) of local kyphosis angle, wedge deformity rate (defined as {Hb-Ha}/Hb × 100, where Ha is the height of the anterior wall of the fractured vertebra and Hb is the height of the posterior wall), and spinal canal stenosis rate (defined as {L_average-L_fracture}/L_average × 100, where L_average = {La+Lb}/2, L_fracture is the anterior-posterior diameter of the spinal canal at the fracture site, La is the diameter at the cephalad vertebra, and Lb is the diameter at the caudal vertebra) (Figures 3A-3C) [9]. Values are reported as mean±standard deviation.

Statistical analyses

One-way Analysis of Variance (ANOVA) with Tukey’s multiple post hoc test was used to compare mean preoperative parameters with those immediately after surgery and before and after FRI removal. A p-value less than 0.05 is considered significant. All statistical analyses were performed using EZR (Saitama Medical Center, Jichi Medical University: Saitama, Japan), which is a graphical user interface for R version 3.5.1 (The R Foundation for Statistical Computing: Vienna, Austria). More precisely, it is a modified version of R Commander designed to add statistical functions frequently used in biostatistics [10].

## Results

Clinical results

Surgery was performed an average of four days after injury. The mean intraoperative blood loss was 30±27 (range: 5-90) g, and the mean operative time was 100±36 (range: 45-188) min. PVP was performed in 16 patients. The extent of fixation was one level above to one level below in 17 patients, and the extent of fixation was extended by one vertebra in three patients with adjacent vertebral fractures. Four patients with preoperative neurological deficits recovered to AIS grade E postoperatively and were able to walk (Table 1).

**Table 1:** Clinical characteristics of the patients. LSC: load sharing classification; PVP: percutaneous vertebroplasty; HA: hydroxyapatite; AIS: American Spinal Injury Association Impairment Scale

Case	Age (years)	Sex	Fracture	Type	LSC score	Time from injury to surgery (day)	Mechanism of injury	PVP (HA)	Fixed range (above-below)	AIS (preoperatively)	AIS (postoperatively)
1	73	M	L2	A3	5	15	Fall from height	Yes	1-1	E	E
2	71	F	L1	A3	6	3	Road traffic accident	No	1-1	D	E
3	25	F	L2	A3	4	3	Snowboarding	No	2-1	E	E
4	48	M	L1	A3	6	4	Skiing	No	1-1	E	E
5	42	F	L1	A3	5	0	Fall from height	No	1-1	D	E
6	28	F	T12	A3	7	2	Fall from height	Yes	1-2	C	E
7	46	M	T12	A3	7	3	Road traffic accident	Yes	1-2	D	E
8	35	M	L1	A3	7	3	Snowboarding	Yes	1-1	E	E
9	37	M	L1	A3	6	0	Fall from height	Yes	1-1	E	E
10	61	M	T12	A3	7	2	Fall of heavy objects	Yes	1-1	E	E
11	63	F	L1	A3	6	2	Fall from height	Yes	1-1	E	E
12	41	M	L1	A3	5	1	Fall from height	Yes	1-1	E	E
13	47	F	L1	A3	6	1	Fall from height	Yes	1-1	E	E
14	41	M	L3	A3	5	6	Road traffic accident	Yes	1-1	E	E
15	65	M	T12	A3	6	5	Fall from height	Yes	1-1	E	E
16	20	M	T11	A3	6	5	Snowboarding	Yes	1-1	E	E
17	23	M	L2	B2	7	4	Fall from height	Yes	1-1	E	E
18	41	M	L1	A3	7	20	Fall from height	Yes	1-1	E	E
19	36	F	L1	A3	5	1	Fall from height	Yes	1-1	E	E
20	33	M	L1	A3	6	1	Fall from height	Yes	1-1	E	E

Complications

No patients had implant failure or required a blood transfusion during surgery. There were no infections, and no additional procedures were needed. None of the patients required routine painkillers at the final follow-up.

Radiologic results

**Figure 4:**
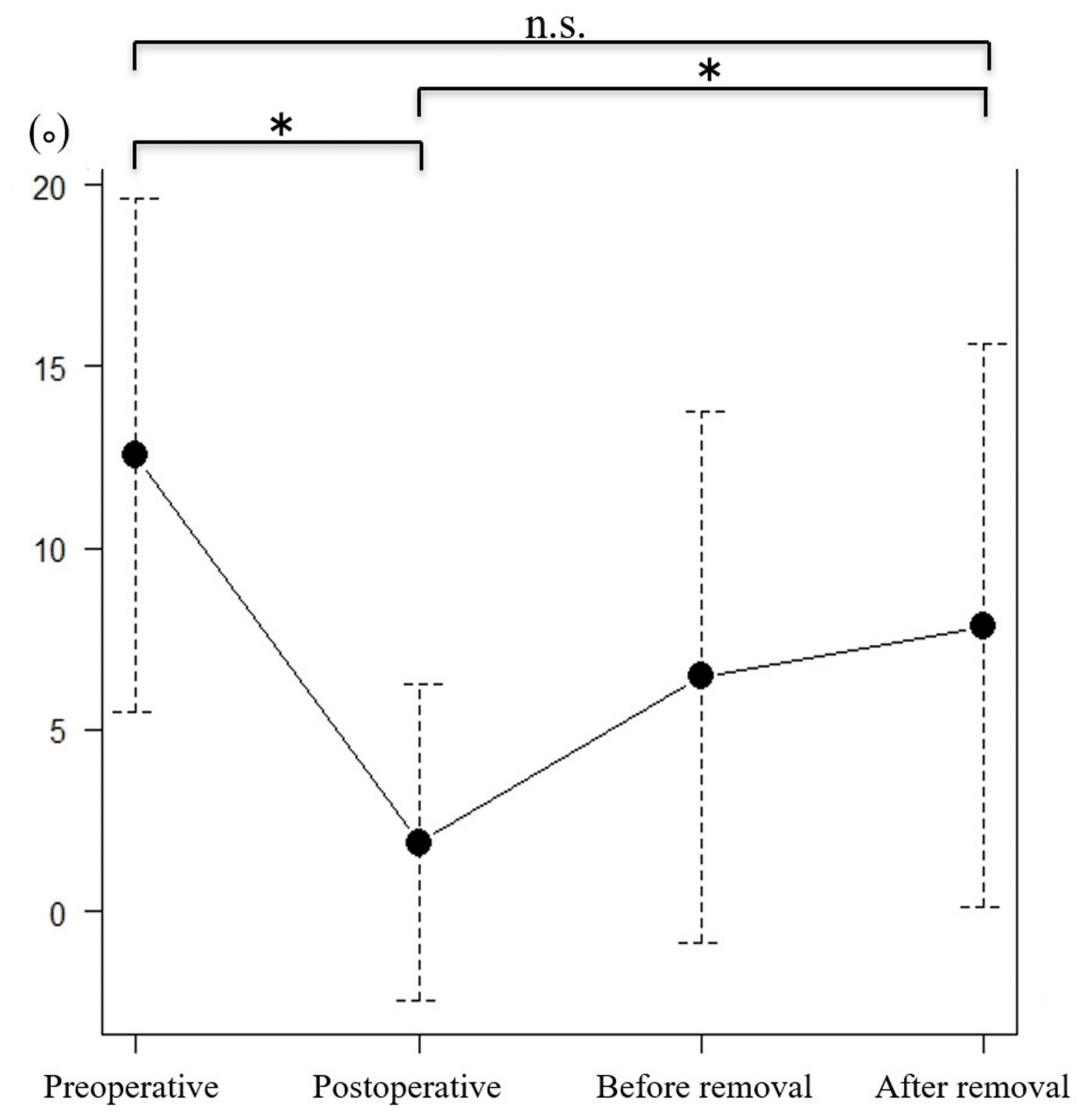
Local kyphosis angle. *P<0.05 was considered statistically significant.

The mean local kyphosis angle was 12.6° preoperatively, 1.9° immediately after surgery, 6.5° before implant removal, and 7.9° after implant removal (Figure 4). Significant improvement was observed between the preoperative and immediate postoperative periods. However, there was also a significant loss of correction after implant removal compared with the immediate postoperative period (mean 6°). The kyphosis angle tended to decrease from the preoperative period to after implant removal, though the change was not statistically significant.

**Figure 5:**
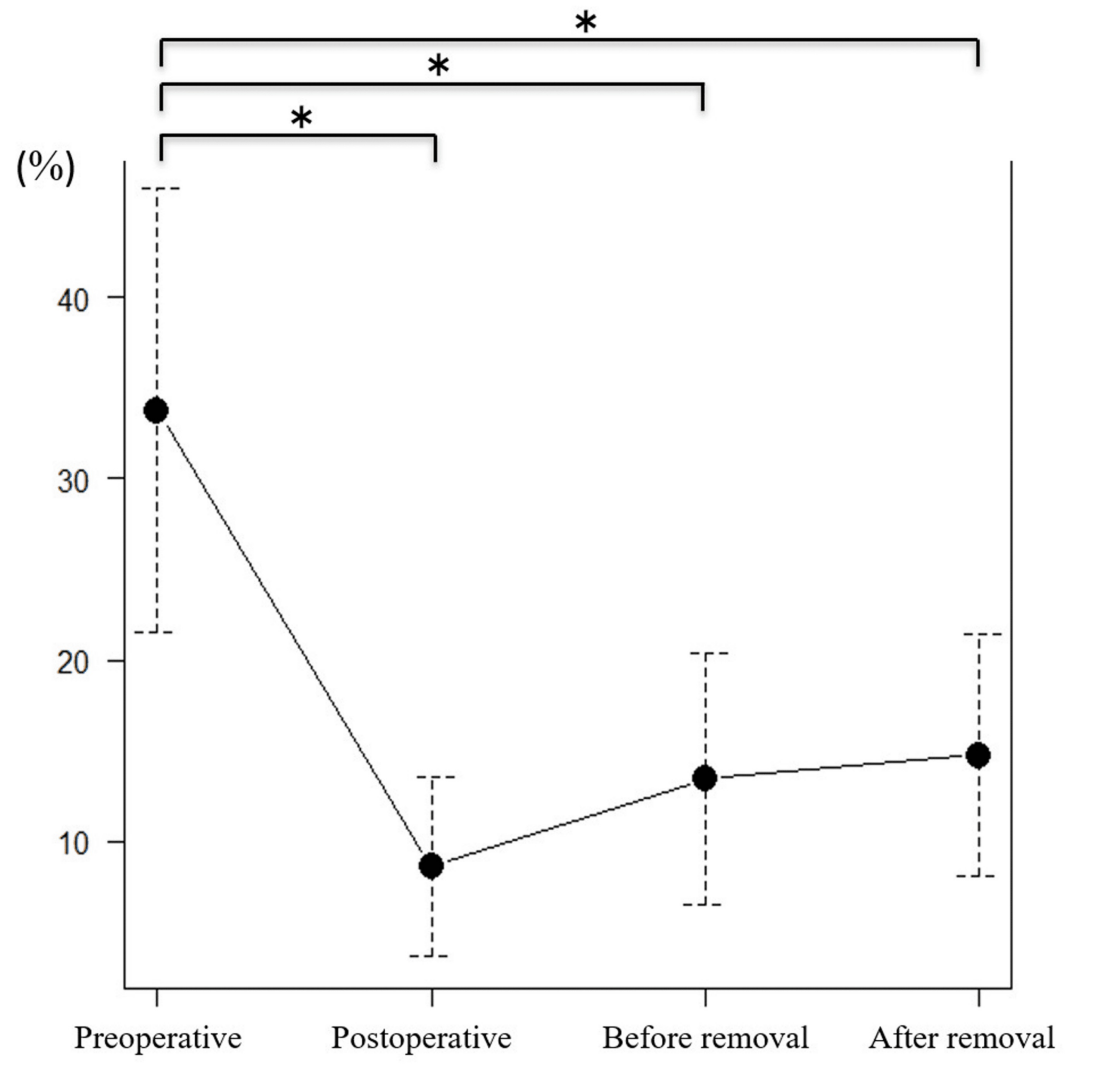
Wedge deformity rate. *P<0.05 was considered statistically significant.

The mean wedge deformity rate showed significant improvement immediately after surgery (33.8% preoperatively, 8.6% immediately after surgery, 13.4% before implant removal, and 14.7% after implant removal; p<0.05) (Figure 5). This improvement was maintained after implant removal (p<0.05). 

**Figure 6:**
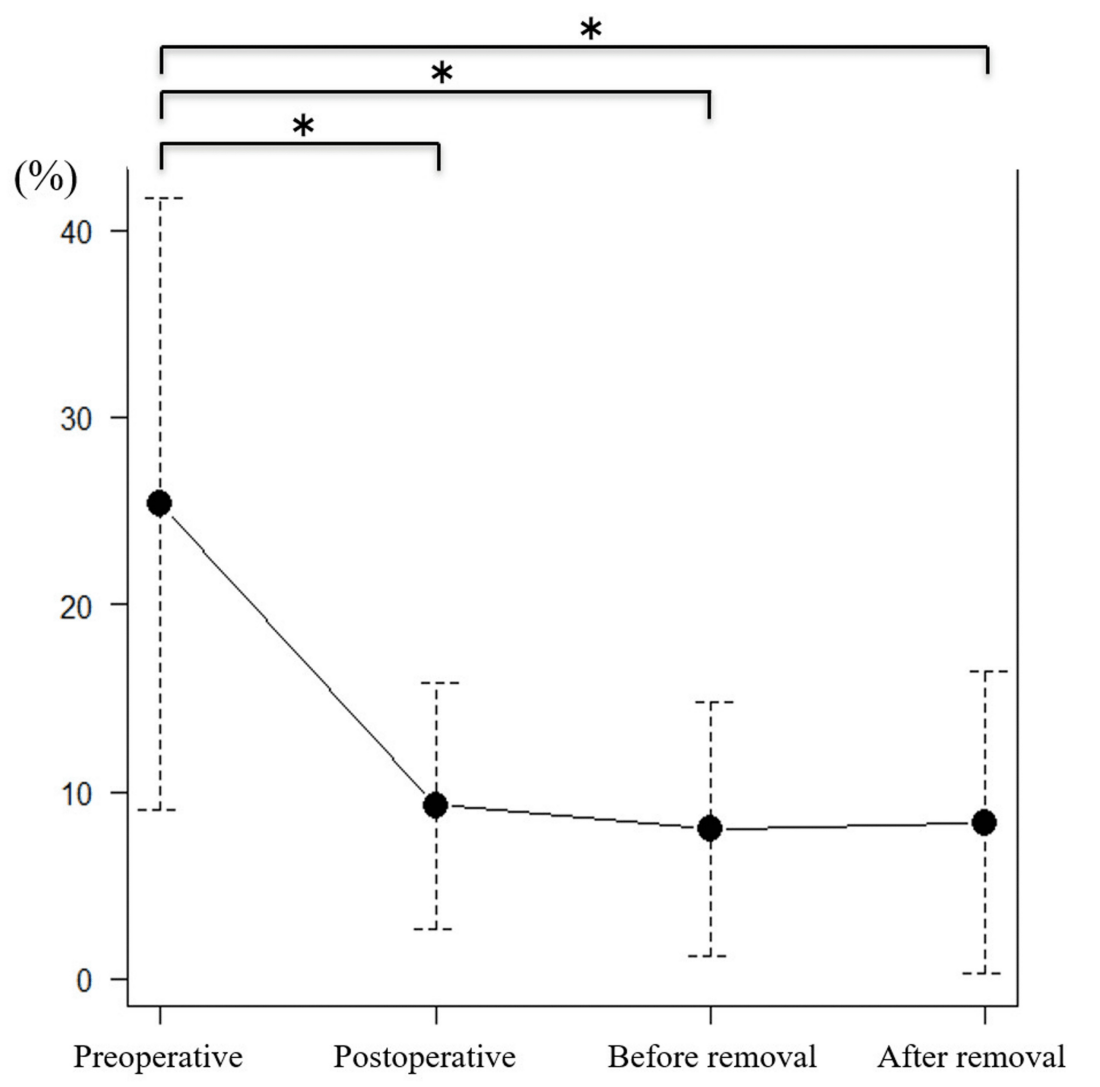
Spinal canal stenosis rate. *P<0.05 was considered statistically significant.

The mean spinal canal stenosis rate also showed significant improvement immediately after surgery (25.3% preoperatively, 9.3% immediately after surgery, 8.0% before implant removal, and 9.4% after implant removal; p<0.05) and was also maintained until after implant removal (Figure 6).

Example case

**Figure 7:**
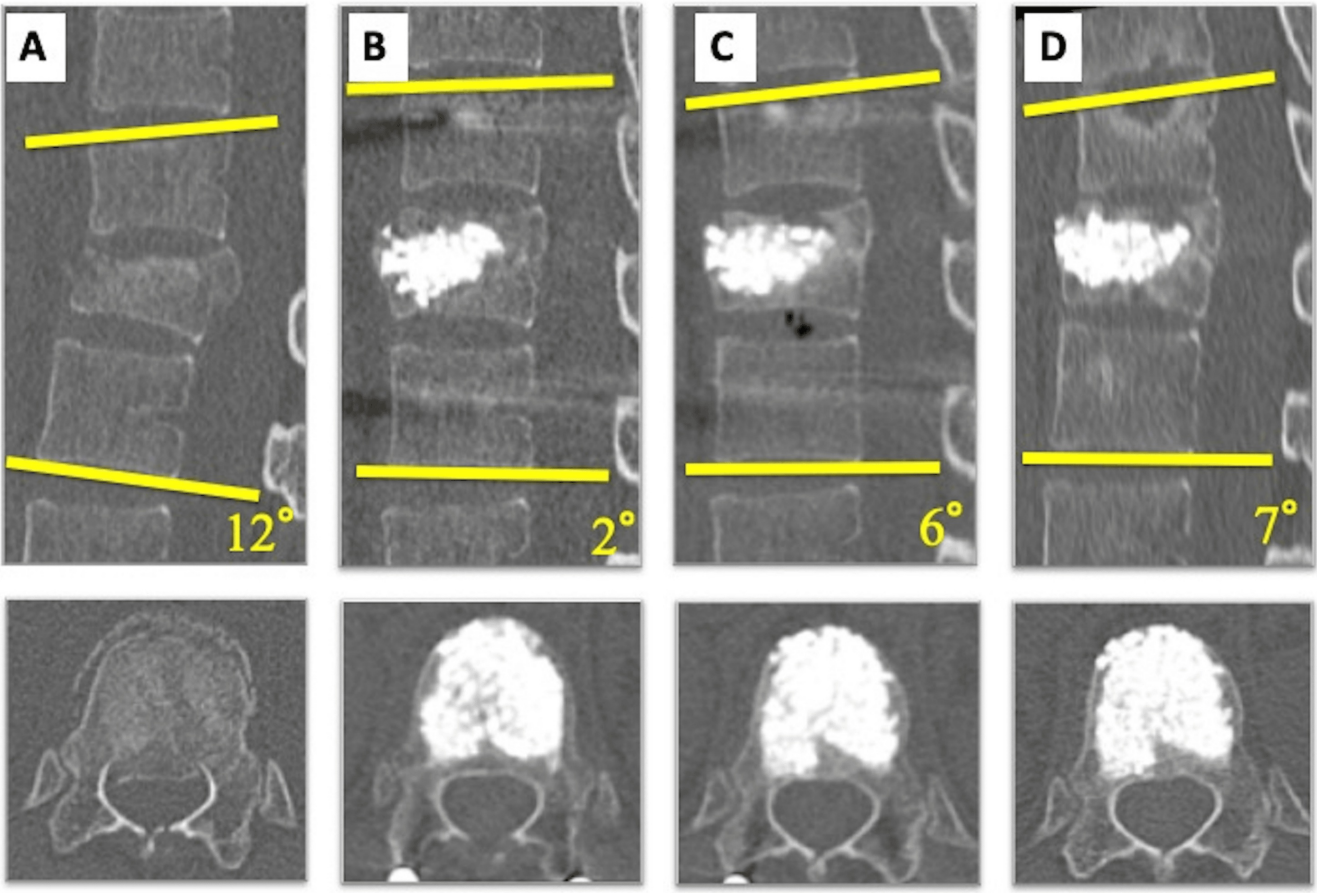
Imaging findings in an example case. (A) Preoperative, (B) postoperative, (C) before implant removal, and (D) after implant removal. A 28-year-old woman sustained a burst fracture of the 12th thoracic vertebra. Immediately after surgery, the local kyphosis angle improved by 2°. During follow-up, there was a slight loss of kyphosis correction, but bony union was achieved, and the spinal canal was remodeled. Local kyphosis angle: (A) 12°, (B) 2°, (C) 6°, (D) 7°. Wedge deformity rate: (A) 57.1%, (B) 3.0%, (C) 10.6%, (D) 10.7%. Spinal canal stenosis rate: (A) 42.5%, (B) 3.9%, (C) 7.2%, (D) 6.0%. This image is created by the author (Shunsuke Kobayashi) of this study.

A 28-year-old woman with schizophrenia fell from a height and sustained a burst fracture of the 12th thoracic vertebra, AO classification A3 (Figures 7A-7D). On admission, she had neurological deficit (AIS grade C), and a CT scan showed a local kyphosis angle of 19°, spinal canal stenosis rate of 42.5%, and wedge deformity rate of 57.1%. Surgery was performed two days after the injury. Operative time was 115 minutes, and intraoperative blood loss was 11 g. Postoperatively, her neurological deficit improved to AIS grade E, and she was able to walk on postoperative day one. She was discharged on postoperative day eight. Immediately after surgery, the local kyphosis angle improved by 2°, the spinal canal stenosis rate by 3.9%, and the wedge deformity rate by 3.0%. During follow-up, there was a slight loss of kyphosis correction, but the bony union was achieved, and the spinal canal was remodeled. The improvements in the spinal canal stenosis and wedge deformity rates were maintained.

## Discussion

We performed percutaneous posterior fixation with PVP in 20 patients with traumatic thoracolumbar vertebral fractures using the S4 Spinal FRI System. In all cases, intraoperative blood loss (30±27 g, range: 5-90 g) and operative time (100±36 min, range: 45-188 min) were acceptable. All patients with preoperative neurological deficits showed improvement.

We believe that this technique combines the advantages of PPSs, such as shorter operative time, earlier surgery, minimal invasiveness to the dorsal muscles, early pain relief, early discharge, and low incidence of surgical site infection [11-14]. In addition, monoaxial screws have the advantage of less loss of correction in comparison with polyiaxial screws [15-18].

McDonough et al. reported an anterior fixation procedure with an intraoperative blood loss of 1,750 mL and an operative time of 295 minutes, making it significantly more invasive than the procedure used in this study [19]. Similarly, Toyone et al. reported single-level posterior intervertebral fixation with Schantz screws, resulting in a blood loss of 162 mL and an operative time of 135 minutes, both higher than in our study. They reported the results of a 10-year follow-up of 12 patients who underwent short-segment fixation without fusion with PVP using an HA block after kyphosis correction with Schantz screws. They reduced bone fractures with ligamentotaxis and removed the implants one year later, achieving good results with a loss of correction of 4°. Lumbar range of motion (flexion-extension) was maintained at an average of 12° (range: 5°-19°), and back pain improved, with no patients requiring regular pain medication. Toyone et al. also highlighted several advantages of avoiding bone grafting, including the absence of graft-related complications, preserved motion, reduced intraoperative blood loss, and shorter operative time [5]. Similarly, our method involved short posterior fixation without bone grafting, offering comparable benefits.

A disadvantage of short posterior fixation for traumatic thoracolumbar vertebral fractures is the loss of correction of local kyphosis. In the present study, there was a significant loss of correction in local kyphosis, with an average of 6° between the immediate postoperative period and after implant removal. Immediately after surgery and before implant removal, the mean kyphosis angle showed a loss of correction of 4.6°, although this was not statistically significant. Disc damage is thought to contribute to the loss of correction after implant removal. Suppression of disc degeneration by good endplate alignment has been reported, and endplate alignment may be important for preventing loss of correction [20]. Additionally, it has been suggested that the reduced physiological load on the discs due to fixation may decrease nutrient supply, leading to disc degeneration [21,22].

Hoppe et al. reported a series of short posterior stabilizations with posterior bone grafting and vertebroplasty using polymethylmethacrylate [23]. The loss of correction after implant removal was 7.3±6.4°, similar to the findings of the present study, with no progression of kyphosis at two to 12 months after implant removal. In that study, the following risk factors for loss of correction were identified: younger age, fracture of the thoracolumbar junction, and higher fracture kyphosis at the time of injury. In this study, all patients were investigated for potential loss of correction due to bent rods or loose rod/screw connections, but no such issues were found in any of the patients.

A disadvantage of the S4 Spinal FRI System is that the connection between the rod and screw must be at a 90° angle. If the direction of PPS insertion is inappropriate, under- or overcorrection may occur. Therefore, the direction of PPS insertion must be carefully considered before surgery. The system is also more technically challenging in kyphotic regions, such as the lower lumbar spine. Consequently, fractures of the thoracolumbar junction with less kyphosis are considered the most appropriate indication for using this system. Further improvements of the S4 Spinal FRI System are needed. It is also important to emphasize that the method investigated in this study is not suitable for osteoporotic vertebral fractures, as the vertebra is subjected to loading during correction. Therefore, the indications for this method should be carefully evaluated, considering preoperative bone quality assessment.

This study has some limitations. The sample size was small, and there was no comparison with a group undergoing posterior fixation alone. These will need to be addressed in future studies. Another limitation is the short follow-up period. One reason for this is that patients without complications after implant removal tend to stop attending follow-up visits. In the future, as disc degeneration progresses with aging, local kyphosis is expected to worsen, making follow-up observation necessary. Additionally, polyaxial screws and PVP were used in a subset of cases as auxiliary techniques. Although neither was central to the surgical strategy, and both were applied in a supportive capacity, their selective use may have introduced some variability. This potential heterogeneity is acknowledged as a limitation of the study.

## Conclusions

This technique combines the advantages of a monoaxial screw with low correction loss and the minimally invasive nature of PPS. In addition, there are no bone graft complications, and the range of motion is preserved with implant removal. This is a useful surgical technique for thoracolumbar vertebral fractures.
